# The role of endobronchial ultrasound‐guided transbronchial needle aspiration liquid‐based cytology in the diagnosis of mediastinal lymphadenopathy

**DOI:** 10.1002/dc.24374

**Published:** 2019-12-28

**Authors:** Chuancai Xu, Lingyan Qin, Wei Lei, Junhong Jiang, Chongjun Ni, Jian'an Huang

**Affiliations:** ^1^ Department of Respiratory Medicine The First Affiliated Hospital of Soochow University Suzhou China; ^2^ Department of Pathology The First Affiliated Hospital of Soochow University Suzhou China

**Keywords:** endobronchial ultrasound (EBUS), liquid‐based cytology, lung cancer, mediastinal lymphadenopathy, transbronchial needle aspiration (TBNA)

## Abstract

**Background:**

Endobronchial ultrasound‐guided transbronchial needle aspiration (EBUS‐TBNA) is a minimally invasive, reliable technique for sampling mediastinal lymph nodes (LNs). Liquid‐based cytology (LBC) is widely used for cervical cancer screening because it provides reliable and feasible results. The present study aimed to evaluate effectiveness of the combination of EBUS‐TBNA and LBC in the diagnosis of mediastinal lymphadenopathy.

**Methods:**

A total of 602 LNs that were retrospectively analyzed were sampled in 442 patients who underwent EBUS‐TBNA between January 2014 and December 2016. The histopathological result of TBNA tissue or cell blocks was considered as the gold standard to evaluate diagnostic utility of LBC and conventional smears (CS) for the diagnosis of mediastinal lymphadenopathy.

**Results:**

Of the 602 LNs, 265 were mediastinal LN metastases from lung cancer, four were lymphoma, and 333 were benign. The sensitivity of LBC and CS in the diagnosis of mediastinal LN metastases from lung cancer was 72.8% and 63%, respectively, and the specificity was 98.5% and 97%, respectively. The positive predictive values for LBC and CS were 97.5% and 94.4%, respectively, whereas the negative predictive values were 82.2% and 76.9%, respectively. The accuracy of LBC and CS was 88% and 83.7%, respectively. The diagnostic value of LBC was significantly higher than that of CS (*P* = .001).

**Conclusions:**

The combination of EBUS‐TBNA and LBC is a highly reliable and feasible procedure that optimizes diagnostic utility for the diagnosis of lung cancer and mediastinal LN staging.

## 1 INTRODUCTION

Recently, endobronchial ultrasound‐guided transbronchial needle aspiration (EBUS‐TBNA) has emerged as a first‐line, minimally invasive procedure for the diagnosis of patients with mediastinal lymphadenopathy.^1^, ^2^ Under guidance of an ultrasound probe, mediastinal lymph node (LN) biopsies can be performed effectively with TBNA. Moreover, EBUS‐TBNA has been demonstrated high sensitivity (92%) and excellent diagnostic accuracy (98%) in the diagnosis and staging of lung cancer.^3^ Hence, current guideline‐recommended EBUS‐TBNA as the first‐line approach for mediastinal LN staging of lung cancer.^4^

Liquid‐based cytology (LBC), as an extensively used cytopathologic technique, is a widely used method for screening cervical cancer because it significantly improved detection rates of cervical cancer and precancerous lesions.^5^ Recently, LBC for non‐gynecological specimens has gained increasing importance due to good fixation and well‐preserved nuclear details,^6^ which got more reliable and feasible results compared with conventional smears (CS). Therefore, the present study aimed to retrospectively evaluate diagnostic utility of EBUS‐TBNA combined with LBC in mediastinal lymphadenopathy.

## 2 PATIENTS AND METHODS

This retrospective study included 442 patients with mediastinal lymphadenopathy who underwent EBUS‐TBNA at the First Affiliated Hospital of Soochow University between January 2014 and December 2016. Chest computed tomography (CT) findings of all the patients showed significant mediastinal LN enlargement with a short‐axis diameter > 1 cm. Patients underwent an electrocardiogram, routine blood tests, and coagulation tests before TBNA were conducted. This study was approved by the Ethics Committee of the First Affiliated Hospital of Soochow University. Written informed consent was obtained from each patient for flexible bronchoscope examination.

**Figure 1:**
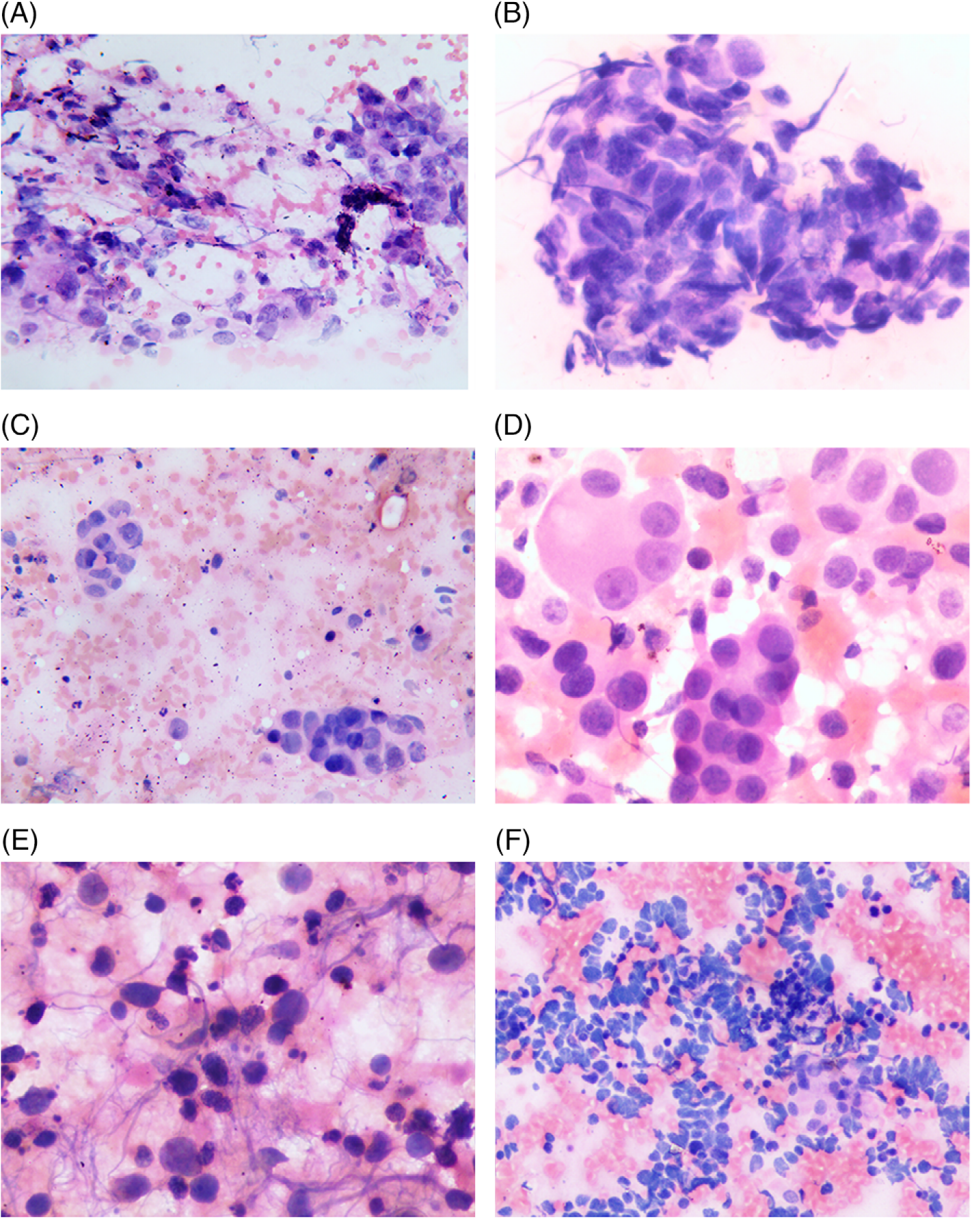
The distribution of tumor cells was uneven on CS of SCC (A). The tumor cells in LBC (B) were more clearly defined and atypical than those on CS of SCC. Compared with CS of adenocarcinoma (C), LBC (D) showed a clearer background. Tumor cells in LBC of SCLC (F) were more atypical than those on CS (E), with necrotic background. Specimens from both CS and LBC were stained with hematoxylin and eosin staining (×40). Abbreviation: CS, conventional smears; SCC, squamous cell carcinoma; LBC, liquid‐based cytology; SCLC, small cell lung cancer [Color figure can be viewed at http://wileyonlinelibrary.com]

The patients fasted for at least 4 hours prior to the procedure. Before commencing the bronchoscopy, local anesthesia was achieved with 2% lidocaine. Some patients also received an intravenous administration of midazolam and (or) fentanyl for conscious sedation. Blood pressure, heart rate, oxygen saturation, electrocardiogram, and consciousness level of the patients were monitored during the procedure. Initially, conventional bronchoscopy was performed, and then convex probe endobronchial ultrasound (BF‐UC260FW, Olympus, Tokyo, Japan) was used for EBUS‐TBNA, with a scanning frequency of 7.5 MHz to detect a target LN and peripheral vasculature. Ultrasound images were processed with a universal endoscopic ultrasound scanner (EU‐ME1, Olympus). Once the target LN was located, a compatible 21 G thin needle (NA‐201SX‐4021, Olympus) was inserted into the working channel of the bronchoscope. Subsequently, the needle was allowed to pierce the airway wall and enter the LN using a jabbing technique under real‐time ultrasound guidance. Each LN station was assessed systematically, and a minimum of three passes per node was performed. Rapid on‐site cytological evaluation was not used. The aspirates were then immersed and rinsed in a vial containing PreservCyt solution (ThinPrep, Hologic, Marlborough, MA, USA). Then, ThinPrep slides were prepared using the ThinPrep 2000 (Hologic, USA) automated slide processor. Part of the aspirates were uniformly spread onto glass slides and fixed with 95% ethanol to make CS slides. The aspirates of next puncture were taken to make CS and LBC in the reversed order. Specimens from both CS and LBC were stained with hematoxylin and eosin (H&E), and an optical microscope was utilized to examine the slides (Figure 1). A positive cytologic result of malignancy was accepted as evidence of cancer. It was considered a cytological negative outcome that no malignancy was seen in the cytologic preparations. In addition, the staining result was considered benign if a few atypical cells were identified but were insufficient for a malignant diagnosis. After obtaining histology samples, the specimens were immediately fixed with 10% formalin, *embedded* in paraffin, sliced, stained with H&E. If adequate tissue was not obtained during the TBNA procedure, we used the aspirates to make cell blocks for a histopathological diagnosis. The final pathology diagnosis of histology samples (including cell blocks) of EBUS‐TBNA was considered as the gold standard. Immunohistochemistry was performed on histology samples or cell blocks to further clarify the classification of lung cancer.

The present study was approved by the Ethics Committee of the First Affiliated Hospital of Soochow University. Signed informed consent was obtained from the patients or guardians.

### 2.1 Statistical analysis

The sensitivity, specificity, accuracy, positive predictive values and negative predictive values of CS and LBC were calculated by using standard formulas. Diagnostic utility of these techniques for adenocarcinoma, small cell lung cancer, and total lung cancer cases was compared using McNemar's test. Fisher's exact test was used in comparing diagnostic utility of LBC with that of CS for squamous cell carcinoma and non‐small cell lung cancer not otherwise specified (NSCLC‐NOS). All statistical analyses were performed using the SPSS statistical software package (SPSS version 19.0, Chicago, USA). *P* < .05 was considered statistically significant.

## 3 RESULTS

A total of 442 patients included in this study comprised 294 men, with a mean age of 62 years (range 19‐89), and 148 women, with a mean age of 58.5 years (range 21‐86). Summary of diagnoses of LNs is presented in more detail in Tables 1, 2, 3. Among all the 602 mediastinal LNs, 521 (86.5%) were diagnosed by EBUS‐TBNA combined with LBC, which was significantly higher than the number diagnosed by CS (490, 81.4%) (*P* < .001) (Table 4).

**Table 1:** Summary of diagnoses of lymph node (LNs) included in the study

Histopathological results	Patients, n (%)	LNs, n (%)
Mediastinal LNs metastasis from lung cancer	195 (44.1)	265 (44)
Lymphoma	2 (0.5)	4 (0.7)
Benign	245 (55.4)	333 (55.3)
Total	442 (100)	602 (100)

**Table 2:** Summary of diagnoses of mediastinal LNs metastasis from lung cancer

Histopathological results	Patients, n (%)	LNs, n (%)
SCC	22 (11.3)	27 (10.2)
ADC	98 (50.3)	129 (48.7)
SCLC	45 (23.1)	68 (25.7)
ADSC	5 (2.6)	5(1.9)
CSCLC and ADC	4 (2.1)	5(1.9)
NSCLC‐NOS	21 (10.8)	31 (11.7)
Total	195 (100)	265 (100)

Abbreviations: ADC, adenocarcinoma; ADSC, adenosquamous carcinoma; CS, conventional smears; CSCLC and ADC, combined small cell lung carcinoma and adenocarcinoma; LN, lymph node. LBC, liquid‐based cytology; NSCLC‐NOS, non‐small cell lung cancer not otherwise specifiedSCC, squamous cell carcinoma; SCLC, small cell lung cancer.

**Table 3:** Summary of diagnoses of benign lymph node (LNs)

Histopathological results	Patients, n (%)	LNs, n (%)
Reactive lymphoid hyperplasia	186 (75.9)	246 (40.9)
Granuloma	50 (20.4)	73 (12)
Tuberculosis	8 (3.3)	13 (2.2)
Fungi	1 (0.04)	1 (0.2)
Total	245 (100)	333 (100)

**Table 4:** Summary of cytological diagnoses of LBC and CS

Histopathological results	LBC results		CS results		
	Malignant, n (%)	Benign, n (%)	Malignant, n (%)	Benign, n (%)	Total, n (%)
Mediastinal LN metastasis from lung cancer	193 (72.8)	72 (27.2)	167 (63)	98 (37)	265 (100)
Lymphoma	0	4 (100)	0	4 (100)	4 (100)
Benign	5 (1.5)	328 (98.5)	10 (3)	323 (97)	333 (100)

Abbreviations: CS, conventional smears; LBC, liquid‐based cytology; LN, lymph node.

In our study, 63 lung cancer patients were smokers, accounting for 32.3% of total lung cancer patients. The majority (54, 85.7%) of lung cancer smokers were men (Table 5). Han's study on the application of LBC in the early diagnosis of lung cancer showed that smokers accounted for approximately one‐third of the total population.^7^

**Table 5:** The number of smokers and nonsmokers of the patients with lung cancer

	Male		Female	
	Smoking	Nonsmoking	Smoking	Nonsmoking
SCC, n (%)	10 (45.5)	4 (18.2)	3 (13.6)	5 (22.7)
ADC, n (%)	20 (20.4)	25 (25.5)	3 (3.1)	50 (51)
SCLC, n (%)	19 (42.2)	12 (26.7)	2 (4.4)	12 (26.7)
ADSC, n (%)	0	4 (80)	1 (20)	0
CSCLC and ADC, n (%)	2 (75)	1 (25)	0	1 (25)
NSCLC‐NOS, n (%)	3 (14.3)	12 (57.1)	0	6 (28.6)
Total, n (%)	54 (27.7)	58 (29.7)	9 (4.6)	74 (37.9)

Abbreviations: ADC, adenocarcinoma; ADSC, adenosquamous carcinoma; CSCLC and ADC, combined small cell lung carcinoma and adenocarcinoma; NSCLC‐NOS, non‐small cell lung cancer not otherwise specified; SCC, squamous cell carcinoma; SCLC, small cell lung cancer.

The sensitivity of LBC and CS in the diagnosis of mediastinal LN metastases from lung cancer was 72.8% and 63%, respectively, and the specificity was 98.5% and 97%, respectively. The positive predictive values for LBC and CS were 97.5% and 94.4%, respectively, whereas the negative predictive values were 82.2% and 76.9%, respectively. McNemar's test revealed a significant difference between the two groups (*P* = 0.001). The sensitivity of LBC and CS to detect LN metastases of squamous cell carcinoma was 63% and 55.6%, respectively, however, no statistically significant difference was observed by Fisher's exact test. McNemar's test showed significant difference in the sensitivity of LBC and CS for the detection of adenocarcinoma (*P* = .027), particularly in small cell lung cancer (*P* = .001) (Table 6).

**Table 6:** Comparison of diagnostic utility between LBC and CS in mediastinal LNs metastasis from lung cancer

Histopathological results	LBC results		CS results			*P*‐value
	Malignant, n (%)	Benign, n (%)	Malignant, n (%)	Benign, n (%)	Total	
SCC	17 (63)	10 (37)	15 (55.6)	12 (44.4)	27	1
ADC	104 (80.6)	25 (19.4)	93 (72.1)	36 (27.9)	129	0.027
SCLC	61 (89.7)	7 (10.3)	46 (67.6)	22 (32.4)	68	0.001
ADSC	2 (40)	3 (60)	2 (40)	3 (60)	5	—
CSCLC and ADC	4 (80)	1 (20)	4 (80)	1 (20)	5	—
NSCLC‐NOS	21 (67.7)	10 (32.3)	22 (71)	9 (29)	31	1
Total	193 (72.8)	72 (27.2)	167 (63)	98 (37)	265	0.001

Abbreviations: ADC, adenocarcinoma; ADSC, adenosquamous carcinoma; CSCLC and ADC, combined small cell lung carcinoma and adenocarcinoma; CS, conventional smears; LBC, liquid‐based cytology; LN, lymph node; NSCLC‐NOS, non‐small cell lung cancer not otherwise specified; SCC, squamous cell carcinoma; SCLC, small cell lung cancer.

## 4 DISCUSSION

EBUS‐TBNA is a highly accurate and safe method for the diagnosis and staging of lung cancer.^4^ However, in clinical practice, due to a variety of reasons, there may be no appropriate tissue samples obtained by TBNA for a diagnosis. Air drying and improper dispersions of cells during the processing of CS remain primary causes of inadequate cytological results.^8^ LBC, which does not require special technical training or skills to collect the material, is a well‐established method for the diagnosis of cervical carcinoma.^9^, ^10^ Over the past few years, the LBC techniques ThinPrep and SurePath have been extensively implemented for the cytological diagnosis of non‐gynecological specimens,^11^ moreover, LBC has been widely used for respiratory tract specimens.^7^ In addition, LBC specimens can be efficiently used for molecular tests when the available material is insufficient to prepare cell blocks.^12^ Kobayashi et al analyzed the difference in cell number, cell morphology, and slide background between CS and LBC samples obtained by TBNA, and then concluded that LBC could be reliably and routinely used in specimens obtained via TBNA.^13^ Fan et al reported the sensitivity of LBC (71.6%) was significantly higher than that of traditional cytology (57.8%) in the diagnosis of lung cancer, and LBC was also found to be more valuable in the diagnosis of small cell lung cancer.^14^ Consistent with these findings, the present study revealed that the diagnostic yield of LBC in identifying mediastinal LN metastases of lung cancer was significantly higher than that of CS. Our study also found that the positive detection rate of small cell lung cancer was significantly higher than that of non‐small cell lung cancer, which may be attributed to cell morphology and the extensive mediastinal LN metastases from small cell lung cancer.

Furthermore, Li et al showed that LBC exhibited a more specific background, well‐preserved cell morphology and the high diagnostic sensitivity (89.6%).^15^ Previous reports also revealed the significantly higher diagnostic sensitivity (95.4%) of LBC in small cell lung cancer compared with that of CS (75.5%). This finding was consistent with ours in which LBC was superior to CS in differentiating benign and malignant cells, particularly small cell lung cancer.

False negatives in the present study occurred probably due to the following reasons: malignant cells were not aspirated or not seen, or misinterpreted as benign. There were preparation artifacts which obscured cytological detail.^16^

Several limitations of our study must also be acknowledged. First, this study was a retrospective analysis and subject to the limitations of the study design, so an information bias was inevitable. Second, in this study, the patients included were all from one single hospital.

In conclusion, the combination of EBUS‐TBNA and LBC is a highly reliable, safe, and feasible procedure that optimizes diagnostic utility for the diagnosis of lung cancer and mediastinal LN staging.

## CONFLICT OF INTEREST

The authors declare that they have no competing interests.

## Data Availability

The datasets used and/or analyzed during the present study are available from the corresponding author on reasonable request.
